# Strategy for Adapting Wine Yeasts for Bioethanol Production

**DOI:** 10.3390/ijms10010385

**Published:** 2009-01-26

**Authors:** Beng Guat Ooi, Kevin R. Lankford

**Affiliations:** Department of Chemistry, Middle Tennessee State University, P.O. Box 68, Murfreesboro, Tennessee, 37132, USA; E-Mail: krl2k@mtsu.edu

**Keywords:** Fermentation, ethanol yield, respiratory deficient mutants, wine yeast, lycorine resistance, mitochondrial DNA

## Abstract

The *Saccharomyces cerevisiae* wine yeast strains 71B-1122 and K1-V1116 were used to derive strains that could tolerate and produce higher ethanol yields. Respiratory-deficient mutants resistant to 500 μg/mL lycorine were isolated. Two mutants, 71B-1122 YEBr L3 and K1-V1116 YEBr L4, were shown to achieve about 10% and 18% improvement in their glucose-to-ethanol conversion efficiency compared to their respective parent strains. The K1-V1116 YEBr L4 in particular can tolerate an ethanol yield of 18.8 ± 0.8% at 3.5 weeks of fermentation and continued to consume most of the sugar until less than 1% glucose was left.

## 1. Introduction

Ethanol is a desirable fuel additive because it allows fuel to burn more cleanly and lowers greenhouse gas emissions. It is cost-effective to blend ethanol into gasoline in view of high crude oil prices in recent years. The use of ethanol as fuel or a fuel additive may have a positive economic impact on the household income, job market, and tax revenue of the United States [1] including reduction in the public health care cost from a variety of health problems related to exposure to harmful pollutants from gasoline emissions [2]. Most of the gasoline fuel in the United States is blended with up to 10% ethanol that is produced from the fermentation of sugar derived mostly from corn [3]. Gasoline blended with 85% ethanol or E85 fuel can also be used in modern flexible fuel vehicles and hence promote the growth of the bioethanol fuel market [4]. Yeast can convert sugar under anaerobic conditions to ethanol in a process referred to as fermentation. A byproduct of fermentation is acetic acid, which lowers ethanol yield and is itself an undesirable component in gasoline fuel formulations. Wine and sherry wine yeasts [5,6] are generally excellent for fermenting sugar because they can produce high ethanol yield with low acetic acids.

The objective of this project is to mutate two different wine yeast strains (71B-1122 and K1-V1116) to form new strains that can produce higher ethanol yields than their parent strains. Two different methods of mutating yeast cells were compared in this study. One method uses ethidium bromide as a mutagen to induce deletions or loss of mitochondrial DNA to create respiratory-deficient *rho*^−^ or *rho*^0^ mutants, respectively [7,8]. The other method uses a high concentration of ethanol to induce respiratory-deficient *rho*^0^ or *rho*^−^ mutants. By allowing the cells to grow in a high concentration of ethanol, the mitochondrial genome can be altered or deleted [6,9]. The mechanism for this is unclear. Ethanol can cause severe damage to yeast mitochondrial membrane but has not been shown to cause chromosomal DNA damage in wild-type yeast cells [10] suggesting that ethanol might have induced mtDNA mutation through membrane damage [6,9].

## 2. Experimental Section

### 2.1. Yeast Strains and Growth Media

**Figure 1.**
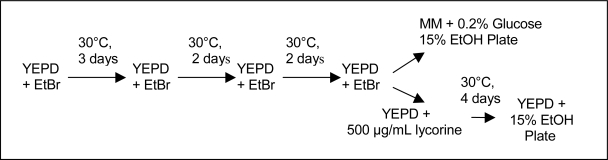
Flow chart of the culture method using ethidium bromide to induce yeast mutation.

**Figure 2.**
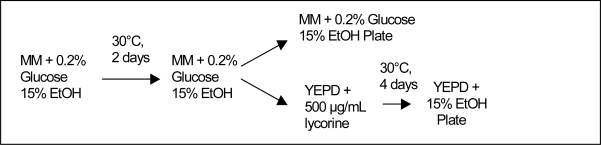
Flow chart showing the culture method of using ethanol to induce yeast mutation.

The wine yeast strains *Saccharomyces cerevisiae* var. *cerevisiae* 71B-1122 and K1-V1116 were Lalvin strains purchased from Grape and Granary (Akron, Ohio, USA). Mutants derived from the ethidium bromide mutation method (Figure 1) were designated YEBr and mutants derived using the exposure to ethanol method (Figure 2) were designated MGEt. Those designated with the letter “L” were also resistant to 500 μg/mL lycorine. Minimal media (MM) was prepared with 0.67% Difco yeast nitrogen base without amino acids (Voigt Global Distribution, Lawrence, Kansas, USA). In the minimal media containing 0.2% glucose, the glucose was added after sterilization. The complete media (YEPD) contained 1% yeast extract, 2% Bacto^®^ peptone, 2% glucose and the media plates contain the corresponding media plus 2% granulated agar. The ethidium bromide solution contained 1 mg/mL of ethidium bromide (Sigma-Aldrich, St. Louis, Missouri, USA) dissolved in deionized water that was filtered through a 0.22 μm pore size, 25 mm diameter GE Cameo syringe filter (Fisher Scientific, Fairlawn, New Jersey, USA).

### 2.2. Culture Methods

The yeast strains (71B-1122 and K1-V1116) were mutated by subjecting the cells to several growth generations (Figure 1) in YEPD containing 25 μg/mL of ethidium bromide (EtBr) under aerobic conditions or growing them in minimal media containing 0.2% glucose, and 15% ethanol under anaerobic conditions (Figure 2). The cell cultures were incubated at 30 °C for numerous growth cycles according to the schedules given in Figures 1 & 2. Following the mutation cycles, the cells were plated onto MM plates containing 0.2% glucose and 15% ethanol while the remaining yeast cells were centrifuged, rinsed with YEPD, and re-suspended in 4.0 mL of YEPD containing 500 μg/mL lycorine [11]. The yeast cells were grown in YEPD/lycorine media for 4 days and shaken at 200 rpm before being transferred to YEPD plates containing 15% ethanol.

### 2.3. Fermentation Method

**Figure 3.**
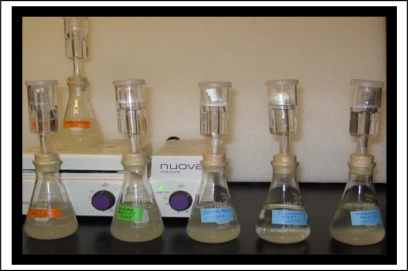
Fermentation setup showing that each flask was sealed with an air lock filled with sterile water to keep the culture under anaerobic conditions. Cultures were stirred daily to re-suspend the settled cells and to remove carbon dioxide from the media.

Overnight pre-inoculum cultures were prepared in YEPD and the optical density (OD) of the culture was determined at the wavelength of 600 nm using a Hitachi U-2000 UV-Vis spectrophotometer. Each aliquot (50 mL) of the media containing 20% glucose and 0.67% nitrogen base was inoculated with 1.2 × 10^8^ cells based on the conversion factor of 0.50 OD being equal to 1 × 10^7^ cells. The flasks were topped with an air lock filled with sterile water to the point where no exchange with ambient air occurred (Figure 3). The fermentation was carried out in triplicate.

The cultures were grown in a Barnstead Lab-line MaxQ 4000 incubator at 25 °C and stirred daily for approximately one minute or until the settled cells were re-suspended. Starting on the seventh day, the amount of glucose left in the fermentation was determined using the Clinitest kit (Fisher Scientific, Fairlawn, New Jersey, USA). When the glucose level in the culture dropped to 1% or less, the cells were removed by centrifugation and the supernatant filtered through a 0.22 μm pore size, 25 mm diameter, GE Cameo syringe filter. In the “teased” fermentation method, the initial culture contained 40 mL of minimal media with 20% glucose. On the seventh day when the glucose level in most of the cultures had dropped to 1 – 2% or less, 10 mL of 60% glucose was added to each culture to a final concentration of 12 – 14 % sugar and the fermentation was allowed to continue for 3 weeks. Samples were collected at 3.5 weeks and at 4 weeks of fermentation for analysis.

### 2.4. Analysis of Ethanol and Acetic Acid

Ethanol and acetic acid concentrations from the fermentation samples were determined using the Agilent Technologies’ 6890 Series II GC (Agilent Technologies, Santa Clara, California, USA) with a flame ionization detector (FID) and a HP-5 30-meter column having 0.25 mm internal diameter and 0.25 μm film thickness. An injection volume of 1 μL, split injection mode with a 20:1 ratio, and helium carrier gas at a flow rate of 1.3 mL/min were used. The GC temperature program had an initial temperature of 40 °C held for 5 minutes followed by a temperature gradient of 6.0 °C/min to 80 °C, held for 1.0 minute, then increased again at the rate of 20 °C/min to 280 °C for a final hold time of 1.0 minute, resulting in the total runtime of 23.67 min. All samples contained 15% (v/v) 2,2,3,3,4,4,5,5-octafluoro-1,6-hexanediol (Oakwood Products, Inc., West Columbia, South Carolina, USA) as an internal standard. Analytical data from triplicate injections were averaged to yield quantitative results based on external calibration of five standard concentrations of ethanol and acetic acid. Analysis of variance (ANOVA) for evaluating the statistical significance of the difference in ethanol yields among the different strains was carried out using the ANOVA feature of the Microsoft Excel program at the significance level or “α value” of 0.05 and 0.01 [12].

### 2.5. Analysis of mtDNA by Gel Electrophoresis

Mitochondrial DNA was prepared using the methods described by Querol and co-workers [13]. The enzyme-digested mitochondrial DNA was separated on 0.5% agarose gel formulated for pulsed-field gel electrophoresis using the TBE buffer (89 mM Tris-borate, 2 mM EDTA, pH 8). The restriction enzymes and the molecular weight markers, namely λ *Hind*III and λ *Eco*RI *Hind*III, were purchased from ProMega Madison, Wisconsin, USA. Gels were stained in TBE buffer containing 1 mg/mL ethidium bromide for 20 minutes.

## 3. Results and Discussion

**Figure 4.**
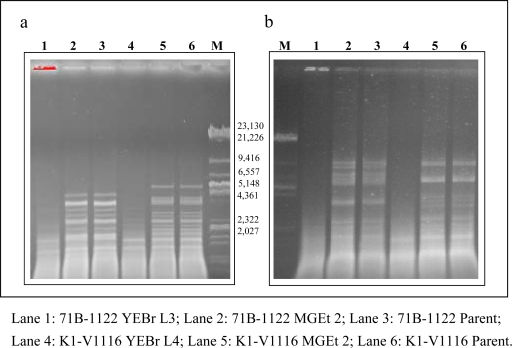
Gel electrophoresis of *Hin*fI (a) and *Rsa*I (b) digestion of DNA extracted from respiratory deficient mutants and parental strains. The molecular weight markers of the λ *Eco*RI and λ *Eco*RI-*Hind*III mix are shown in Lane M. The fragment sizes are listed in base pair units beside the lanes. The other lanes contained the DNA restriction digestion fragments from the following strains.

A total of six new strains were isolated in this study. Four of the strains were resistant to 500 μg/mL of the alkaloid, lycorine. It has been reported that respiratory deficient mutants exhibit different degrees of sensitivity to lycorine. The mutants resistant to the lycorine at a concentration of 500–600 μg/mL could potentially be *rho*^0^ mutants [11,14]. This phenotype could have resulted from a lycorine-resistant nuclear mutation that is associated with both nuclear and mtDNA replication [15] or it could be due to the cellular compensation for the mitochondrial condition through “retrograde regulation” [16] by overexpressing their nuclear *RTG* genes, which also conferred the resistance to lycorine [14]. Massardo *et al*. have demonstrated that the resistance to lycorine depends on the DNA mitochondrial deletion and strains with high DNA mitochondrial deletions show an intermediate resistance [17]. In order to fully distinguish between *rho*^0^ and *rho*^−^ mutants with up to 90–95% mtDNA deletions, further analysis by fluorescence microscopy with 4’,6-diamidino-2-phenylindole (DAPI) staining of yeast cells needs to be conducted [17]. The mutants K1-V1116 YEBr L4 and 71B-1122 YEBr L3 were selected from lycorine-YEPD plates after several growth passages through YEPD media containing ethidium bromide (Figure 1). These strains were also able to grow on media containing 15% ethanol. Gel electrophoresis of *Rsa*I and *Hin*fI digestion of total DNA extracted from K1-V1116 YEBr L4 and 71B-1122 YEBr L3 showed that the mtDNA were deleted from both strains (Figure 4).Ethidium bromide, which intercalates into double stranded DNA, was able to cause respiratory–deficient mutations by causing DNA damage or by affecting DNA synthesis [18]. The two mutant strains, K1-V1116 MGEt 2 and 71B-1122 MGEt 2, after being selected via several growth generations in media containing 15% ethanol, still retained similar mtDNA restriction pattern as their corresponding parental strains [5]. Although earlier investigators had postulated that alcohol could induce a high degree of polymorphisms in mtDNA [6,19], the DNA restriction analysis of these strains did not reveal major mtDNA polymorphisms, suggesting that ethanol might have induced only small lesions on these mtDNA or that ethanol might not be mutagenic to mtDNA as indicated previously [6]. Therefore, the influence of ethanol on mtDNA loss in the other lycorine-resistant strains, 71B-1122 MGEt L3 and 71B-1122 MGEt L4 isolated in this study, might be related to another mechanism.

Ibeas and Jimenez (1997) confirmed the earlier findings [20–22] that mitochondrial genome plays an important role in ethanol tolerance when flor yeasts mitochondria transferred into laboratory strain conferred ethanol tolerance on the recipient laboratory strain [6]. In this study, wine yeasts that generally produced about 13.5% ethanol were used to derive mitochondrial mutants that could potentially tolerate and produce a higher ethanol yield than their parental strains.

**Figure 5.**
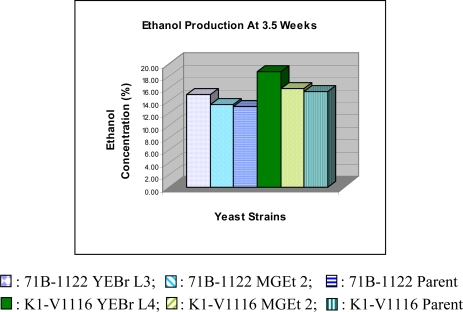
Comparison of Ethanol Yield from Mutant and Parental Strains at 3.5 Weeks of Fermentation. The fermentation samples were from the following yeast strains.

The strains in this study were initially tested in a batch culture containing 20% glucose in minimal media. The mutants and parent control strains consumed almost all of the sugar within 7 – 11 days to produce 11.5 – 12.5% ethanol (Table 1) and acetic acid in the range of 0.09 – 0.11%. However, in the “teased” fermentation where more glucose was added to the cultures after seven days and the fermentation continued until 3.5 weeks, the ethanol yield increased significantly for K1-V1116 YEBr L4 to about 18.8% (Table 2). While the K1-V1116 parent strains were able to produce about 15.5 ± 0.5% ethanol, the 71B-1122 parent strains could only produce around 13.1 ± 0.7% ethanol in 3.5 weeks. The fermentations did not progress further even though there was still glucose left in the media. The amount left was around 4 – 6% for the K1-V1116 strain and greater than 6% for the 71B-1122 strain at the end of the fourth week. However, the K1-V1116 YEBr L4 mutant with deleted mitochondrial genome consistently consumed the sugar at the fastest rate and produced the highest ethanol yield. At 3.5 weeks, it produced 18.8 ± 0.8% ethanol and continued to consume most of the sugar until about less than 1% glucose was left at the end of the fourth week. This showed that the K1-V1116 YEBr L4 mutant was able to tolerate a significantly higher level of ethanol compared to its parent strain (Figure 5). The K1-V1116 MGEt 2 strain also has improved ethanol yield compared to its parent strain. Its ethanol yield was 16.0 ± 0.6% at 3.5 weeks and consumed more glucose until about 2 – 4% was left at the end of the fourth week. The 71B-1122 YEBr L3 carried out the fermentation at a slower rate and it also had a greater tolerance for ethanol compared to its parent strain (Figure 5). Its ethanol yield at 3.5 weeks was about 15.0 ± 0.3% but the amount of glucose left remained at around 4 – 6% until the end of the fourth week. The ANOVA statistics reveals that there are differences in the ethanol production among the six strains. The *F* value of 33.53, in comparison to the critical value of 3.11 for *F*_0.05,5,12_, indicates that the *F* value is significant at the 95% confidence level. Therefore, this corroborates the fact that there are differences in ethanol yields among the strains tested. All the strains produced ethanol with very minimal yield of less than 0.11% acetic acid, suggesting that the acetic acid synthesis was not affected by the mutation or the ethanol production.

## 4. Conclusions

The expedient method of exposing the yeast cells to 25 μg/mL ethidium bromide followed by 500 µg/mL lycorine solution generated mutants from both the 71B-1122 and K1-V1116 strains that were capable of producing and tolerating higher ethanol yields than their parent strains. The yeast strains of 71B-1122 YEBr L3 and K1-V1116 YEBr L4 showed a 10% and 18% improvement in their glucose-to-ethanol conversion efficiency compared to their parent strains, respectively. The MGEt 2 mutant derived from the K1-V1116 parent strain through the ethanol exposure method was also tolerant to a higher level of ethanol and gave a higher ethanol yield of 16.0 ± 0.6% compared to the 15.0 ± 0.3% yield produced by the 71B-1122 YEBr L3 strain that was derived from strain 71B-1122. The control strains 71B-1122 and K1-V1116 stopped fermenting the glucose when the ethanol levels reached 13.1 ± 0.7% and 15.5 ± 0.5% respectively.

The benefit of selecting for petite mitochondria-deficient strains despite their slower growth rate compared to the normal cells was the derivation of new yeast strains with higher ethanol tolerance. This is feasible because high mitochondrial genome polymorphism has been shown to confer ethanol tolerance in flor and other *Saccharomyces* yeast [6,21,22]. Both the wine industry and the bioethanol industry for fuel production have exploited the low acetic acid yields of selected yeast strains. The lower acetic acid makes the taste of the wine more acceptable. In the fermentative process of bioethanol production, the lower acetic acid is associated with higher ethanol yields and results in a fuel with lower emission of acetaldehyde, a suspected carcinogen. The K1-V1116 wine yeast performed better in ethanol production and was also more tolerant to ethanol than the 71B-1122 wine yeast strain. Hence, the mutants derived from K1-V1116 also performed better than those derived from the 71B-1122. The mutation methods carried out in this study could be applied to the *Saccharomyces* flor yeast strains [6] that generally produced greater than 15% ethanol instead of the wine yeast strains that generally yield about 13.5% ethanol under well-controlled industrial enological conditions. This study demonstrated that it is possible to generate respiratory-deficient mutants that will produce a higher ethanol yield than their original strains while maintaining the low acetic acid yield. This method of creating new strains with improved ethanol yields was simple, rapid, and provides an alternative to genetically engineered yeast strains. A comparative analysis of the growth rates of the mutants and their mutation stabilities will lead to a more complete understanding of their characteristics.

## Figures and Tables

**Table 1.** Average concentrations of ethanol and acetic acid produced in batch fermentation.

Yeast Strains	71B-1122 YEBr L3	71B-1122 MGEt 2	71B-1122 MGEt L3	71B-1122 MGEt L4	71B-1122 Parent	K1-V1116 YEBr L4	K1-V1116 MGEt 2	K1-V1116 Parent
Ethanol % (v/v)	12.3 ± 1.0	11.6 ± 0.7	11.2 ± 0.6	11.7 ± 1.4	12.1 ± 0.9	12.5 ± 1.5	12.3 ± 1.8	12.4 ± 0.6
Acetic Acid % (v/v)	0.09 ± 0.02	0.10 ± 0.01	0.09 ± 0.01	0.10 ± 0.02	0.11 ± 0.02	0.07 ± 0.00	0.11 ± 0.01	0.11 ± 0.01

Fermentation was carried out in batch culture containing 20% glucose in minimal media. Samples were collected for analysis only after most of the sugar was consumed by the yeast i.e. when the sugar test indicated trace or less than 1% sugar. The theoretical yield for converting 50 mL of 20% glucose (10 grams) to ethanol is about 13% ethanol.

**Table 2.** Average concentration of ethanol produced in “teased” fermentation.

Yeast Strains	71B-1122 YEBr L3	71B-1122 MGEt 2	71B-1122 Parent	K1-V1116 YEBr L4	K1-V1116 MGEt 2	K1-V1116 Parent	ANOVA F value^a^
Ethanol % (v/v) (3.5 weeks)	15.0 ± 0.3	13.4 ± 0.8	13.1 ± 0.7	18.8 ± 0.8	16.0 ± 0.6	15.5 ± 0.5	33.53
Ethanol % (v/v) (4 weeks)	15.4 ± 0.4	12.2 ± 1.5	11.8 ± 1.2	19.4 ± 0.5	16.6 ± 1.0	15.2 ± 0.4	27.02

Fermentation was extended from 1 week to 4 weeks by adding more glucose to each culture. Those strains that could tolerate higher ethanol levels continued to ferment the glucose and increased their ethanol yield significantly compared to levels shown in Table 1. The theoretical yield for converting 14 grams of glucose in 50 mL of “teased” fermentation sample to ethanol is about 18.2% ethanol.

a The critical *F* value (α value = 0.05) for all samples was 3.11.
